# Involvement of Yeast HSP90 Isoforms in Response to Stress and Cell Death Induced by Acetic Acid

**DOI:** 10.1371/journal.pone.0071294

**Published:** 2013-08-15

**Authors:** Alexandra Silva, Belém Sampaio-Marques, Ângela Fernandes, Laura Carreto, Fernando Rodrigues, Martin Holcik, Manuel A. S. Santos, Paula Ludovico

**Affiliations:** 1 Life and Health Sciences Research Institute, School of Health Sciences, University of Minho, Braga, Portugal; 2 Life and Health Sciences Research Institute/3B’s - PT Government Associate Laboratory, Braga/Guimarães, Portugal; 3 Department of Biology and Centre d’Enseignement de la Statistique Appliquée à la Médecine, University of Aveiro, Aveiro, Portugal; 4 Apoptosis Research Centre, Children’s Hospital of Eastern Ontario Research Institute, Ottawa, Ontario, Canada; Enzo Life Sciences, Inc., United States of America

## Abstract

Acetic acid-induced apoptosis in yeast is accompanied by an impairment of the general protein synthesis machinery, yet paradoxically also by the up-regulation of the two isoforms of the heat shock protein 90 (HSP90) chaperone family, Hsc82p and Hsp82p. Herein, we show that impairment of cap-dependent translation initiation induced by acetic acid is caused by the phosphorylation and inactivation of eIF2α by Gcn2p kinase. A microarray analysis of polysome-associated mRNAs engaged in translation in acetic acid challenged cells further revealed that HSP90 mRNAs are over-represented in this polysome fraction suggesting preferential translation of HSP90 upon acetic acid treatment. The relevance of HSP90 isoform translation during programmed cell death (PCD) was unveiled using genetic and pharmacological abrogation of HSP90, which suggests opposing roles for HSP90 isoforms in cell survival and death. Hsc82p appears to promote survival and its deletion leads to necrotic cell death, while Hsp82p is a pro-death molecule involved in acetic acid-induced apoptosis. Therefore, HSP90 isoforms have distinct roles in the control of cell fate during PCD and their selective translation regulates cellular response to acetic acid stress.

## Introduction

Yeast cells respond to stress in multiple ways ranging from activation of pathways that promote survival to those eliciting programmed cell death (PCD). The cell’s initial response to an imposed stress is driven towards survival and the defense against and recovery from the insult. Nonetheless, the persistency of the noxious unresolved stimulus eventually activates death signaling pathways. Hydrogen peroxide (H_2_O_2_) and acetic acid are among the stress agents whose yeast cellular responses are best characterized [1]–[3]. Our previous work has shown that acetic acid-induced apoptosis in yeast cells is characterized by the impairment of general protein synthesis, yet it is paradoxically associated with the up-regulation of the two isoforms of the heat shock protein 90 (HSP90) [4]. However, the biological relevance of these alterations in regulating stress recovery or the progression to PCD and the molecular mechanism driving HSP90 induction are still unknown. Mammalian HSP90 chaperones are a family of highly conserved proteins with crucial functions in various cellular processes including signal transduction, protein folding and degradation and protein trafficking between sub-cellular compartments [5]–[8]. HSP90 proteins play important anti-apoptotic functions [9]; however, recent reports also suggested their involvement in necrotic mechanisms [8]–[13] since the HSP90 inhibitors prevent necrosis induced by Fas- and tumor-necrosis factor receptor 1 [10], [11]. Most likely, the different HSP90 isoforms, their selective post-translational modifications and client proteins could explain the dual role of HSP90 in cell survival and death. In yeast, two genes, *HSC82* and *HSP82*, encode Hsp90 proteins, which are 97% identical [14], but are apparently differently regulated [15]. In physiological conditions, Hsc82p is present at 20-fold greater levels than Hsp82p; however, upon heat shock *HSP82* expression is induced more than 20-fold [16]. Among the different HSP90 functions, these chaperones are also involved in yeast translational control [17], [18] and necrotic cell death [12], [19]. Despite this, the translation control of *HSP90* mRNAs and their impact on yeast cell death has not yet been addressed.

Translation control allows cells to reduce protein synthesis and prevent continued gene expression under potentially error prone conditions, save cellular energy and allow the reprogramming of existing mRNAs and proteins, conferring on cells the plasticity needed to deal with stress. Thus, translational control has been identified as an important biological determinant of cell fate through its tight regulation of stress responses and PCD [20]–[23]. Nonetheless, translational control of yeast PCD is still poorly understood. The deletion of the *LSM4* gene, involved in yeast mRNA decay, was shown to inhibit mRNA decapping leading to an increase in mRNA stability and ultimately triggering a caspase-dependent apoptotic process [24]–[26]. Furthermore, an extensive degradation of ribosomal RNAs has been described during H_2_O_2_- and acetic acid-induced apoptosis [27]. In the presence of H_2_O_2_, the degradation of ribosomal RNAs is correlated with decreased global translation mediated by the phosphorylation of eIF2α by Gcn2p kinase (the amino acid control kinase) [28]. Acetic acid treatment also decreased the levels of translation factors eIF4A, eEF1A, eEF2 and eEF3A, a condition known to induce a severe amino acid-starvation response [4].

In this study, we demonstrate the existence of a translational reprogramming of the HSP90 isoforms, particularly of *HSP82* during the progression of acetic acid-induced cell death. In addition, we show that HSP90 isoforms could play antagonistic roles in the cell death process.

## Materials and Methods

### Strains, Media and Treatments

*Saccharomyces cerevisiae* BY4742 (*MATα his3*Δ1 *leu2*Δ0 *lys2*Δ0 *ura3*Δ0) strain and its isogenic derivatives Δ*gcn2,* Δ*hsc82 and* Δ*hsp82* (EUROSCARF) were used. For acetic acid treatment, *S. cerevisiae* cells were grown until the middle exponential phase in liquid YPD medium containing glucose (2%, w/v), yeast extract (0.5%, w/v) and peptone (1%, w/v). Cells were harvested, resuspended in fresh medium (pH 3.0) and incubated at 26°C for 1 h. Then cells were resuspended (10^8^ cells/mL) in YPD fresh medium (pH 3.0) followed by the addition of 160, 180, 195 or 210 mM acetic acid and incubation for 15, 30, 60, 120 and 200 min at 26°C with stirring (150 r.p.m.), with or without the presence of 100 µM of 17-allylaminogeldanamycin (17AAG), a specific inhibitor of HSP90 through interaction with the N-terminal ATPase domain of HSP90 [29]. After the 200 min treatment, approximately 300 cells were spread on YPD agar plates and viability was determined by counting colony forming units (C.F.U.) after 2 days of incubation at 26°C. Cells were also harvested for further studies described below.

### HSP90 Expression

To express *HSC82* and *HSP82* in the respective mutant strains, *HSC82* and *HSP82* were amplified by PCR (*HSC82* primers 5′-GGATCCATGGCTGGTGAAACTT-3′ and 5′-ATCGATTTAAAGATCTTCTTCAGA-3, *HSP82* primers 5′-GGATCCGGAAGCTTGATGACAGA-3′ and 5′-ATCGATTTAAAGATCTTCTTCAGAA-3′) using genomic DNA and cloned by homologous recombination in the pUG35 plasmid (CEN, *URA3*, *MET25* promoter, EUROSCARF) generating pUG35*HSC82* and pUG35*HSP82*. Δ*hsc82* cells were transformed with pUG35*HSC82* and Δ*hsp82* cells with pUG35*HSP82*.

### Polysome Profile Analysis

Yeast cells were grown to middle exponential phase in liquid YPD medium, harvested, resuspended in fresh YPD medium (pH 3.0) and incubated as described above. Five min before cells being harvested, cycloheximide (CHX, Sigma-Aldrich) was added to a final concentration of 100 µg/mL to block protein synthesis elongation, cells were collected, washed twice and disrupted using lysis buffer [20 mM Tris at pH 8.0, 140 mM KCl, 1.5 mM MgCl2, 0.5 mM DTT, 100 µg/mL CHX, 1 mg/mL heparin and 1% Triton X-100] and glass beads (Sigma-Aldrich). 40 A_280 nm_ units of cell lysate were loaded onto 11 mL 15–50% sucrose gradients containing 20 mM Tris-HCl at pH 8.0, 140 mM KCl, 5 mM MgCl2, 0.5 mM DTT, 100 µg/mL CHX and 500 µg/mL heparin. Gradients were centrifuged using a SW41 rotor (Beckman Coulter) at 35000 r.p.m. for 2 h and 45 min at 4°C. Polysome profiles were visualized by monitoring RNA absorbance at 254 nm using a Bio-Rad Biologic LP system adapted for this use. Monosome and polysome fractions of the gradient were collected into tubes containing 8 M guanidine-HCl and stored at –80°C. In order to obtain sufficient quantity of mRNA for microarray analysis, identical fractions were pooled into a single tube.

### Polysome mRNA Preparation

Monosome and polysome fraction mRNA was precipitated with 85% (v/v) ethanol, extracted using phenol: chloroform and precipitated, first with 1.5 M lithium chloride (LiCl) for removing any residual heparin, and then with 100% ethanol plus 110 mM sodium acetate, pH 5.3.

### Quantitative mRNA Expression

Quantitative mRNA expression analysis was performed according to the MIQE (Minimum information for publication of quantitative real-time PCR Experiments) guidelines [30]. RNA was isolated from monosome and polysome fractions as described above. RNA extraction from total cellular extracts was carried out as previously described [30]. qPCR was used to measure the mRNA transcripts of the *HSC82*, *HSP82*, *TIF4632* and *CLN3* genes. Three reference genes (*ACT1*-actin, *PDA1*-alpha subunit of pyruvate dehydrogenase and *TDH2*-isoform 2 of glyceraldehyde-3-phosphate dehydrogenase) were selected due to their stable expression and were tested under the same experimental conditions allowing expression normalization. Primers for qPCR were constructed, by in silico analysis, using Beacon Designer 7.90 software (PremierBiosoft International), and are listed in Table 1. Total RNA was reverse-transcribed into cDNA using the iScript™ cDNA synthesis kit (Bio-Rad), qPCR was performed using the SsoFast Evagreen Supermix™ kit (Bio-Rad) and processed according to the manufacturer’s instructions in a CFX96™ Real Time System (Bio-Rad). A blank (no template control) was also incorporated in each assay. The thermocycling program consisted of one hold at 95°C for 1 min, followed by 39 cycles of 15 min at 95°C, 20 s at 57°C or 60°C and 20 s at 72°C. After completion of these cycles, melting-curve data were collected to verify qPCR specificity, contamination and the absence of primer dimers. The qPCR efficiency of each primer pair (Eff in Table 1) was evaluated by the dilution series method using a mix of sample cDNAs as the template and was determined from calibration curves using the formula 10^(−1/slope)^. Relative expression levels were determined with efficiency correction, which considers differences in the efficiencies between target and reference genes, using the gene expression module of the CFX manager Software (Bio-Rad).

**Table 1:** List of primers for quantification of mRNA expression by qPCR and their efficiency (Eff).

Gene	Primer sequences	Eff (%)
*HSC82*	F: CCGGTGAATCTCTAAAGGCA	96.3
	R: ATCAATTGGGTCGGTCAAGA	
*HSP82*	F: GAGTTGACGAAGGTGGTGCT	97.9
	R: ATGCAAAGGAAGTTGGTTCG	
*TIF4632*	F: TCCGAGGAGACATTAGAGTCCG	99.1
	R: ACCGAACCTTCAAGAGTTGCC	
*CLN3*	F: GCCCCTTTGGAAGCTTTCATT	98.4
	R: TGGCACCCAATTTGATCTCG	
*ACT1*	F: GATCATTGCTCCTCCAGAA	98.2
	R: ACTTGTGGTGAACGATAGAT	
*TDH2*	F: CCGCTGAAGGTAAGTTGA	95.1
	R: CGAAGATGGAAGAGTTAGAGT	
*PDA1*	F: TGACGAACAAGTTGAATTAGC	94.8
	R: TCTTAGGGTTGGAGTTTCTG	

### Microarray Analysis

The microarray analysis was performed according to the MIAME (Minimal Information About a Microarray Experiment) guidelines [31]. Hybridization and sample imaging/quantification was performed at the RNA Biology laboratory of the University of Aveiro. To study *S. cerevisiae* mRNA expression during acetic acid treatment, we used Yeast (V2) Gene Expression Microarray, 8×15 K arrays (V2: G4813A, Agilent Technologies). These are eight-plex arrays that allow for the examination of mRNA expression of ∼6,300 genes from eight samples. The layout provides for three probes per target, the values of which we averaged to obtain a sample value. After polysome RNA extraction, as described above, RNA quantity was assessed using NanoDrop ND-1000 (Thermo Fisher Scientific). RNA quality was assessed by determining the 28S/18S ratio using a Bioanalyzer 2100 (Agilent Technologies). To synthesize targets for hybridization, we began with 200 ng of polysome RNA and used the One-Color Microarray-Based Gene Expression Analysis (Low Input Quick Amp Labeling) kit (Agilent Technologies) to perform the reverse transcription, cDNA transcription and cRNA labelling with Cyanine 3-CTP. Before hybridization, free dye was removed using RNeasy mini spin columns (Qiagen), and the efficiency of cRNA synthesis and dye incorporation (labelling efficiency in pmol Cy3/µg cRNA) was measured by spectrophotometry (NanoDrop 1000). Each hybridization was carried out using 600 ng of Cyanine 3-labelled cRNAs and the Yeast Gene Expression Microarray (Agilent Technologies), for 17 h at 65°C, in an Agilent hybridization oven. Microarrays were scanned using the Agilent DNA Microarrays Scanner G2565AA and raw data was extracted using the Agilent feature extraction protocol GE1_105_Dec08 (PerkinElmer).

Using the normalized M values (log2 ratios) obtained, statistical differences were calculated using the t-test. Differentially expressed genes were identified for a value cut-off of p<0.001. Heatmaps and clustering of genes were carried out using MeV software [32]. Functional analysis of expression data obtained was done using Expander software (Algorithms in Computational Genomics group, Blavatnik School of Computer Science, Tel Aviv University) [33] and the *Saccharomyces* Genome Database GO Term Mapper (db.yeastgenome.org/cgi-bin/SGD/GO/goTermMapper). The data sets are publicly available at ArrayExpress (accession number E-MEXP-3570).

### Immunoblot Analysis

For immunoblot analysis, untreated or acetic acid-treated cells (195 mM) were collected and disrupted using glass beads in lysis buffer [1% v/v Triton X-100, 120 mM NaCl, 50 mM Tris-HCl pH 7.4, 2 mM EDTA, 10% v/v Glycerol, 1 mM PMSF and Complete Mini protease inhibitor cocktail (Roche Applied Science)]. From total protein extracts, 40 µg (100 µg in the protein synthesis analysis assay) were resolved on a 12% SDS-polyacrylamide gel and transferred to an nitrocellulose membrane (Amersham, GE Healthcare) over 90 min at 100 V. Membranes were then blocked with 5% non-fat dried milk in T-TBS (150 mM NaCl, 20 mM Tris–HCl, 0.1% Tween-20, pH 7.5) for 1 h, washed three times with T-TBS and probed with polyclonal rabbit Ser51 phosphorylated eIF2α antibody (1∶2000, Upstate, Merck KGaA), polyclonal rat anti-Tif4631/2p (eIF4G) (1∶1000, kindly provided by Michael Altmann), polyclonal rabbit anti-Eft1/2p (1∶15000), polyclonal rabbit anti-Tef1/2p (1∶15000, both antibodies were kindly supplied by Prof. T.G. Kinzy), polyclonal rabbit anti-Tif1p/2p (1∶15000, kindly supplied by Prof. M. Montero-Lomelí), monoclonal rat anti-HSP90 (1∶1000, Merck Millipore) and polyclonal goat anti-actin (1∶5000, kindly provided by Campbell Gourlay). HRP-conjugated anti-rabbit, anti-rat and anti-goat IgG secondary antibody were used, at a dilution of 1∶5000 and detected by enhanced chemiluminescence.

### TUNEL Assay

DNA strand breaks were assessed by a TUNEL assay with the In situ Cell Death Detection Kit, POD (Roche Applied Science). Briefly, as previously described [1], yeast cells were initially fixed with 3.7% (w/v) formaldehyde followed by digestion of the cell wall with lyticase. After preparation of cytospins, the slides were rinsed with PBS, incubated in permeabilization solution (0.1%, v/v, Triton X-100 and 0.1%, w/v, sodium citrate) for 3 min on ice, rinsed twice with PBS, and incubated with 10 µL of TUNEL reaction mixture (terminal deoxynucleotidyl transferase and FITC-dUTP) for 60 min at 37°C [4]. Finally, the slides were rinsed three times with PBS and a coverslip was mounted with a drop of anti-fading agent Vectashield (Molecular Probes) and with 2 µL of 50 µg/mL propidium iodide (PI, Molecular Probes) solution in Tris buffer (10 mM, pH 7.0) with MgCl_2_ (5 mM) and RNase (0.5 µg/mL). Cells were visualized with an Olympus PlanApo 60×/oil objective, with a numerical aperture of 1.42.

### Flow Cytometry Analysis of Plasma Membrane Integrity

The plasma membrane integrity was analysed using propidium iodide (PI, Molecular Probes) (50 µg/mL in PBS). Cells were harvested after 200 min of acetic acid treatment and PI was added for 20 min at 37°C, washed once with 0.5 mL PBS and resuspended in 0.5 mL PBS. The PI signals were measured using FACSCaliber2 ﬂow cytometer (BD-Biosciences) with a 488 nm excitation laser. Signals from 30000 cells/sample were captured in FL3 (>670 nm) at a ﬂow rate of 1000 cells/s. Data collected with the FACSCaliber2 ﬂow cytometer were processed with FlowJo 7.6 software (TreeStar Inc.).

### Statistical Analysis

The arithmetic means of at least three independent assays for the comparison of cell survival rates and mRNA relative expression are presented with standard deviation with a 95% confidence value. Statistical significance was determined using *t*-test or two-way ANOVA applying Bonferroni correction. A *p*-value of less than 0.05 was considered as a significant difference.

## Results

### Impairment of Global Translation in Acetic Acid Challenged Cells is Mediated by Alterations in the Translational Apparatus

**Figure 1:**
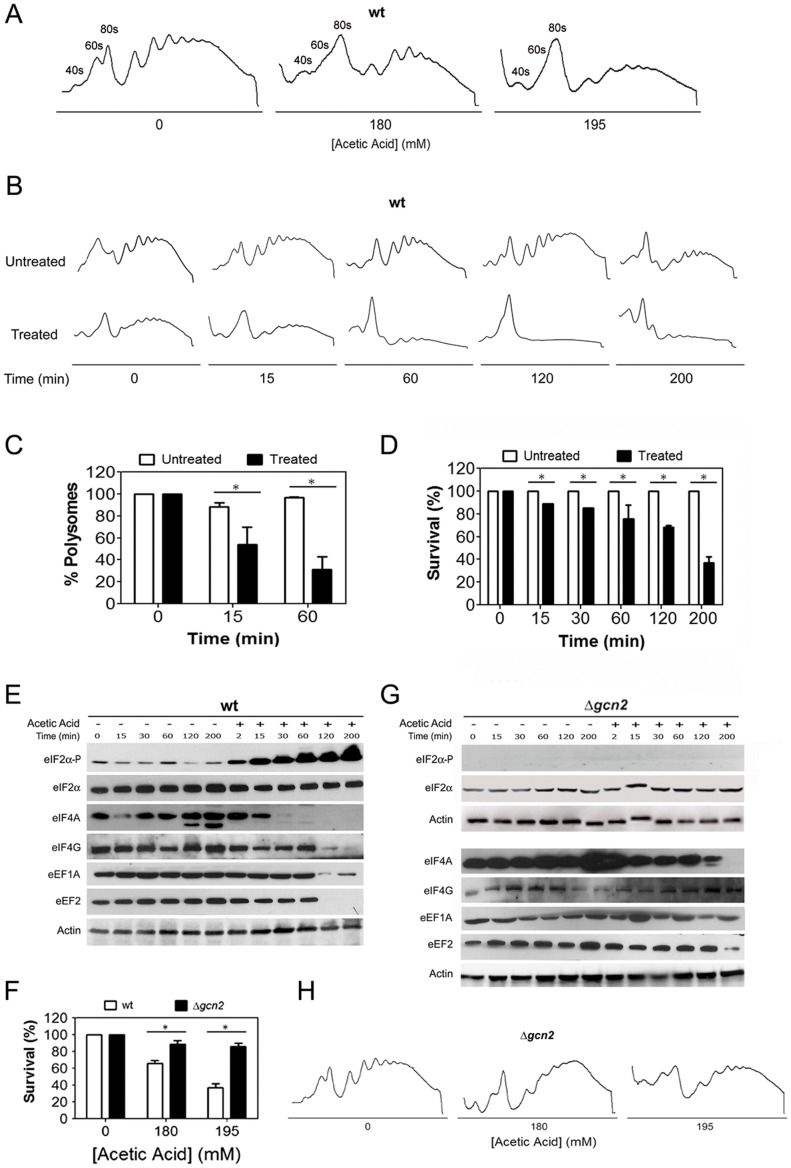
Impairment of global translation during acetic acid treatment is mediated by translation machinery alterations. (A) Polysome profile of wild-type yeast cells treated with the indicated concentrations of acetic acid for 15 min. The peaks containing the small (40S) and large ribosomal subunit (60S), or free ribosomes (80S) are indicated. (B) Time-course analysis of the polysome profiles of wild-type cells untreated or treated with 195 mM of acetic acid for 15, 60, 120 or 200 min. (C) Analysis of the percentage of total mRNA present in the polysome fractions of polysome profiles of wild-type cells at 0, 15 or 60 min of 195 mM of acetic acid treatment (white bars correspond to control untreated cells and black bars to acetic acid treated cells; *p<0.05 versus wild-type untreated cells, t-test, n = 3). (D) Kinetic analysis of wild-type cells survival for 200 min of acetic acid treatment (195 mM) (white bars correspond to control untreated cells and black bars to acetic acid treated cells; *p<0.05 versus wild-type untreated cells, t-test, n = 3). Immunoblot kinetic analysis of eIF2α (Sui2p) phosphorylation levels, and expression levels of translation factors eIF2α (Sui2p), eIF4A (Tif1/2p), eIF4G (Tif4631/2p), eEF1A (Tef1/2p) and eEF2 (Eft1/2p) in (E) wild-type (wt) and (G) *GCN2* deleted *Saccharomyces cerevisiae* cells untreated or treated with 195 mM of acetic acid. Actin was used as loading control. (F) Comparison of the survival rate of wild-type and *GCN2* deleted *S. cerevisiae* cells upon treatment with the indicated concentrations of acetic acid (white bars correspond to wild-type cells and black bars to *GCN2* deleted cells; *p<0.05 versus wild-type cells, t-test, n = 3). (H) Polysome profile of *GCN2*-disrupted yeast cells treated with the indicated concentrations of acetic acid for 15 min.

We have previously shown that induction of apoptosis by acetic acid in yeast cells results in severe attenuation of translation [4]. To further elucidate the mechanism of these alterations, we performed a polysome profile analysis of wild-type cells treated with two different acetic acid concentrations (180 and 195 mM). This analysis revealed an impairment of global translation in acetic acid treated cells, as reflected by the decrease of the polysome fractions (corresponding to the mRNAs actively engaged in translation) and the increase of the monosome fractions (corresponding to the free ribosomal subunits) (Figure 1A). This effect was dependent on the acetic acid concentration tested as cells treated with the higher concentration (195 mM) displayed a more pronounced decrease of the polysome fractions (Figure 1A).

The impact of acetic acid treatment on translation efficiency was investigated by a kinetic analysis of polysome profile changes throughout the 200 min of treatment with 195 mM acetic acid (Figure. 1B and 1C), as well as by the kinetic analysis of cell viability (Figure 1D). This concentration of acetic acid induced a progressive decline of survival and prolonged exposure resulted in 60% loss of proliferative capacity (Figure 1D). The analysis of the polysome profiles showed a progressive decrease in polysomes of acetic acid-treated cells during the first 60 min of treatment that plateaued and was maintained for the duration of the acetic acid treatment (Figure 1B and 1C). As expected, the loss of polysomes was accompanied by the corresponding increase in the monosome fraction at all-time points (Figure 1B).

The observation of global translation impairment upon acetic acid treatment prompted us to investigate factors involved in the initiation and elongation steps of translation. Thus, a kinetic analysis of the levels of the translation initiation factors 4A (eIF4A/Tif1/2p) and 4G (eIF4G/Tif4631/2p) (components of eIF4F initiation complex), and of the elongation factors 1A (eEF1A/Tef1/2p) and 2 (eEF2/Eft1/2p), previously shown in our proteomic analysis to be decreased upon acetic acid challenge [4], was performed. The phosphorylation status of the translation initiation factor 2α (eIF2α/Sui2p) was also examined due to its well known role in translation regulation [34]. Immunoblot analysis revealed that after 15 min of acetic acid treatment the impairment of translation was correlated with alterations in the levels and phosphorylation status of translation initiation factors (Figure 1E). An increase in the levels of phosphorylated eIF2α, and a loss of eIF4A suggested impairment in the assembly of the ternary complex and in the function of the cap-binding complex eIF4F, which results in attenuation of canonical translation initiation at early points of acetic acid treatment. In contrast, the decrease in the levels of the elongation factor eEF1A and the loss of the elongation factor eEF2 and of the initiation factor eIF4G were observed only at 120 min (Figure 1E). The congruent decrease of these translation factors is associated with the almost complete collapse of the polysome profiles observed at 120 min (Figure 1B). The differential loss of initiation factors combined with the knowledge that eIF4G plays a role in yeast and mammalian cap-independent translation [35], [36] suggest that selective translation may be occurring during acetic acid treatment. Nonetheless, further studies are required to understand the mechanisms of translation during acetic acid-induced apoptosis.

Gcn2p is the only kinase known to be responsible for the phosphorylation of eIF2α in yeast [37]–[39]. The observed increase in the levels of eIF2α phosphorylation (Figure 1E) led us to study the role of Gcn2p in the regulation of translation and cell survival during acetic acid treatment. Deletion of *GCN2* resulted in a high resistance phenotype to acetic acid treatment (Figure 1F), which was associated with an abrogation of eIF2α phosphorylation (Figure 1G). Ablation of *GCN2* also prevented polysome collapse (Figure 1H) and significantly attenuated the loss of translation factors (Figure 1G). These observations indicate that treatment of cells with acetic acid leads to an impairment of global translation that is dependent on Gcn2p kinase. Disruption of *GCN2* restores translational competence and renders cells resistant to acetic acid. These data suggest a link between the control of translation and yeast cell death induced by acetic acid.

### Microarray Analysis of mRNAs Translated during Acetic Acid Treatment

**Figure 2:**
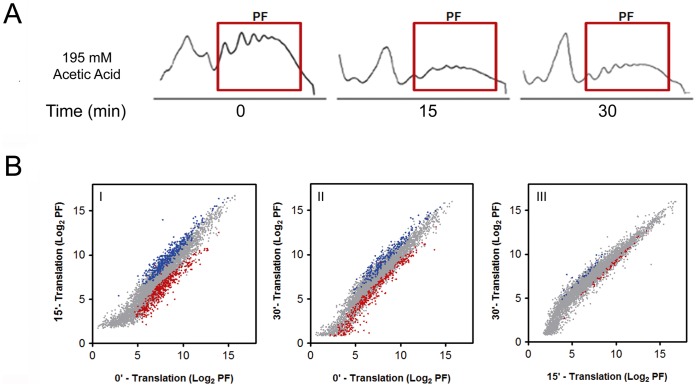
Microarray analysis of mRNAs translated during acetic acid treatment. (A) Polysome fractions (PF) from the polysome profiles of wild-type cells at 0, 15 or 30 min of acetic acid (195 mM) treatment collected for microarray and qPCR analysis. (B) Graphical representations of the translational microarray data normalized by the levels of hybridized mRNAs from the same amount of total polysome mRNAs altered from 0 to 15 min (I), 0 to 30 min (II) and 15 to 30 min (III) of acetic acid treatment normalized by the amount of mRNA in polysome fractions at each time point. The mRNAs in polysome fractions at 15 min or 30 min of acetic acid treatment (log2 PF) have been plotted against the mRNAs in polysome fractions at 0 min of acetic acid treatment (control). Translationally regulated mRNAs (having increased association with polysomes in blue and decreased association with polysomes in red) at 15 min or 30 min of acetic acid treatment have been highlighted in the cross-dot plots.

The data presented above show that although translation is partially inhibited immediately following acetic acid treatment, the complete collapse of polysomes and the loss of eIF4G, eEF1A, and eEF2 occurs only after prolonged exposure to acetic acid. In addition, we have previously shown that the expression of some proteins is increased during acetic acid treatment [4], suggesting that acetic acid treated cells are capable of selective translation. To identify the transcripts that might be translated under these conditions we performed microarray profiling of polysome-associated mRNAs of cells treated with acetic acid for 15 or 30 min (Figure 2). Using a 2-fold cut-off to identify selectively regulated mRNAs, we were able to detect 323 (Figure 2B-I - blue dots) and 132 (Figure 2B-I – red dots) mRNAs whose association with the polysomes increased and decreased, respectively, after 15 min of acetic acid treatment, corresponding to 5.1% and 2.1% of the total number of mRNAs analysed (6392 genes in the array). After 30 min of treatment the polysome association of 165 mRNAs increased (Figure 2B-II - blue dots) and of 176 mRNAs decreased (Figure 2B-II - red dots) when compared to time zero. The altered mRNAs at 15 and 30 min are presented in tables S1 and S2, respectively, and are part of the ArrayExpress database (accession number E-MEXP-3570). These mRNAs correspond to 2.6% and 2.7% of the total number of the mRNAs associated with the polysomes, respectively. The comparison of the mRNAs altered from 15 to 30 min identified 28 mRNAs (Figure 2B-III - blue dots) whose polysome association increased and 71 mRNAs whose polysome association decreased (Figure 2B-III - red dots) from 15 to 30 min of acetic acid treatment, corresponding to 0.65% and 1.11% of the total number of mRNAs analysed.

**Figure 3:**
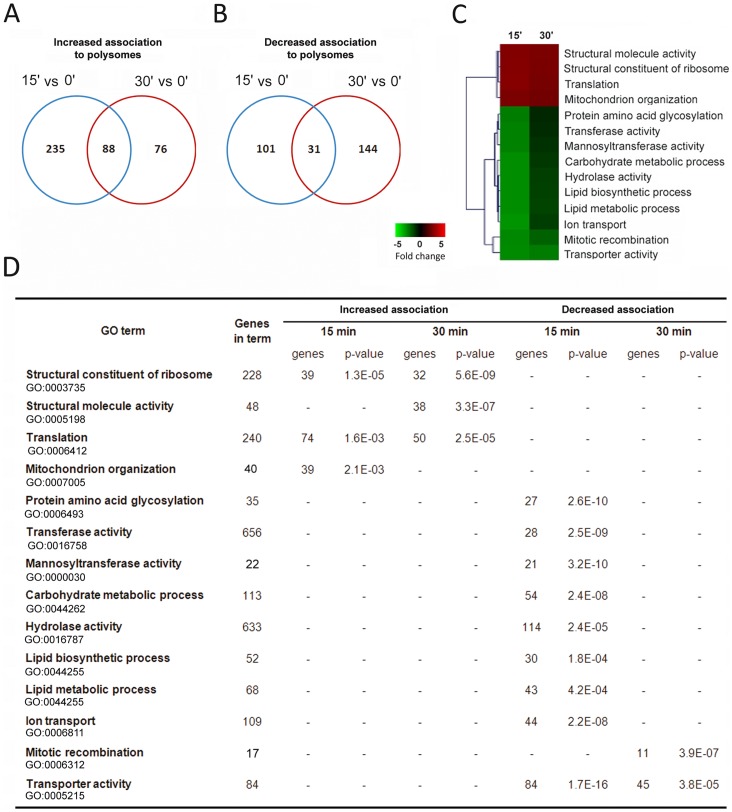
Analysis of the mRNAs whose polysome association was increased after acetic acid treatment. Overlap of mRNAs with (A) increased and (B) decreased association with polysomes at 15 min or 30 min of acetic acid treatment when compared with control (0 min). (C) Summary of GO terms of the mRNAs altered in the yeast cells upon 15 and 30 min of acetic acid treatment. Heatmaps and clustering of genes were carried out using MeV software [32]. Each colour square represents the average expression level of the genes annotated with the corresponding GO term for each condition. (D) Functional analysis of the mRNAs altered in the microarray analysis of yeast cells upon 15 or 30 min of acetic acid treatment was performed using the Expander software TANGO tool (Algorithms in Computational Genomics group, Blavatnik School of Computer Science, Tel Aviv University, Tel Aviv, Israel) [33] and the *Saccharomyces* Genome Database GO Term Mapper (db.yeastgenome.org/cgi-bin/SGD/GO/goTermMapper).

**Figure 4:**
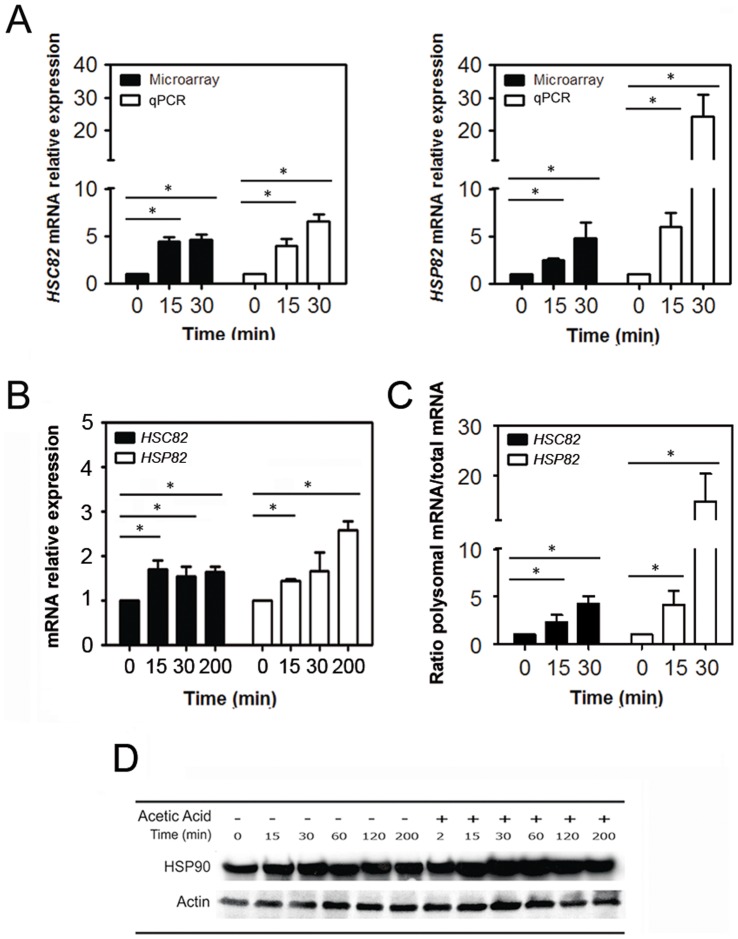
HSP90 isoforms are selectively translated during acetic acid treatment. (A) Comparison of microarray data with qPCR analysis of *HSC82* and *HSP82* mRNA expression in polysome fractions upon 15 or 30 min of acetic acid treatment (black bars correspond to microarray and white bars to qPCR results; *p<0.05 versus time 0 min, *t*-test, n = 3). (B) qPCR analysis of *HSC82* and *HSP82* expression in total mRNA of yeast cells after acetic acid treatment (195 mM) for 0, 15, 30 or 200 min (black bars correspond to *HSC82* mRNA and white bars to *HSP82* mRNA; *p<0.05 versus time 0 min, *t*-test, n = 3). (C) Ratio between *HSC82* and *HSP82* mRNAs associated with polysomes and their presence in total mRNA of yeast cells after acetic acid treatment (195 mM) for 0, 15 or 30 min (black bars correspond to *HSC82* mRNA and white bars to *HSP82* mRNA). (D) Immunoblot kinetic analysis of HSP90 proteins levels in wild-type *Saccharomyces cerevisiae* cells after 0, 15, 30, 60, 120 or 200 min of treatment with acetic acid (195 mM).

A Venn diagram comparison of mRNAs with altered polysome association at 15 and 30 min of acetic acid treatment revealed that 88 mRNAs sustained the gradual increase while 31 mRNAs maintained their lower ribosomal association pattern (Figure 3A and 3B). These results showed that while the polysome association of a small group of mRNAs is maintained from 15 to 30 min of treatment (88 mRNAs), the majority of mRNAs with altered polysome association at each time point is different, suggesting a role for selective translation in the cellular response to stress induced by acetic acid. In addition, the data indicated that despite changes in the abundance and status of translation initiation factors, during acetic acid treatment, translation of specific mRNAs still occurs. Quantitative real time-PCR (qPCR) verifications were performed on six mRNAs in the total polysome fractions used to perform the microarray analysis. The results obtained confirm the increased association with polysomes of molecular chaperones HSP90, namely *HSC82* and *HSP82* (Figure 4A), *TIF34* and *CAF20* mRNAs and the decreased association with polysomes of *COX3* and *CIT2* mRNAs (Figure S1) at 15 and 30 min of incubation with acetic acid.

Microarray results were further analysed and grouped into functional categories using the Expander software TANGO tool and the *Saccharomyces* Genome Database GO Term Mapper (db.yeastgenome.org/cgi-bin/SGD/GO/goTermMapper). Fifteen min of incubation with acetic acid increased the polysome association of mRNAs encoding genes involved in mitochondrial organisation, translation and several structural constituents of the ribosomes (Figure 3C and 3D). In contrast, mRNAs whose translation was reduced at 15 min encode proteins that participate in different metabolic processes such as transport, lipid and carbohydrate metabolism (Figure 3C and 3D). Functional analysis of the mRNAs whose polysome association was increased at 30 min of acetic acid treatment revealed functional groups restricted to cellular functions related to the ribosome structure and regulation of the translation process (Figure 3C and 3D).

### Translation of HSP90 Isoforms is Favoured during Acetic Acid Treatment

We noted that *HSC82* and *HSP82*, two components of the HSP90 chaperone complex, were among the mRNAs that displayed sustained association with polysomes in response to acetic acid treatment, suggesting a role for HSP90 chaperones in acetic acid response. qPCR analysis also confirmed the up-regulation of molecular chaperones HSP90, namely *HSC82* and *HSP82* (Figure 4A) and that both isoforms were enriched in polysomal fractions isolated from yeast cells challenged with acetic acid for 15 and 30 min (Figure S2). Although the correlation between microarrays and qPCR is usually good, higher levels of mRNA expression were observed for *HSP82*, but not for *HSC82*, by qPCR (Figure 4A). Furthermore, qPCR analysis of *HSC82* and *HSP82* total cellular mRNA levels revealed an increased expression at 15 and 30 min, the same time points of the polysomes analysis (Figure 4B). The incremental increase in the ratio between *HSC82* and *HSP82* mRNAs associated with polysomes and their presence in total mRNA of yeast cells after acetic acid treatment (Figure 4C), also reflects that the increase in mRNAs associated with polysomal fractions is not only due to transcription regulation but also to selective translational control. In addition, although immunoblot analysis does not allow the discrimination between Hsc82p and Hsp82p isoforms, we observed a progressive increase of HSP90 levels throughout the treatment with acetic acid (Figure 4D) which is in accordance with our previous proteomic results showing the increase of Hsc82p and Hsp82p protein levels in acetic acid treated cells [4]. Taken together these results showed that the expression of *HSC82* and *HSP82* mRNAs is enhanced during the acetic acid treatment at the level of mRNA transcription and translation, and this occurs despite significant attenuation of global translation observed under these conditions.

### HSP90 Isoforms Display Distinct and Antagonist Functions in Response to Acetic Acid

**Figure 5:**
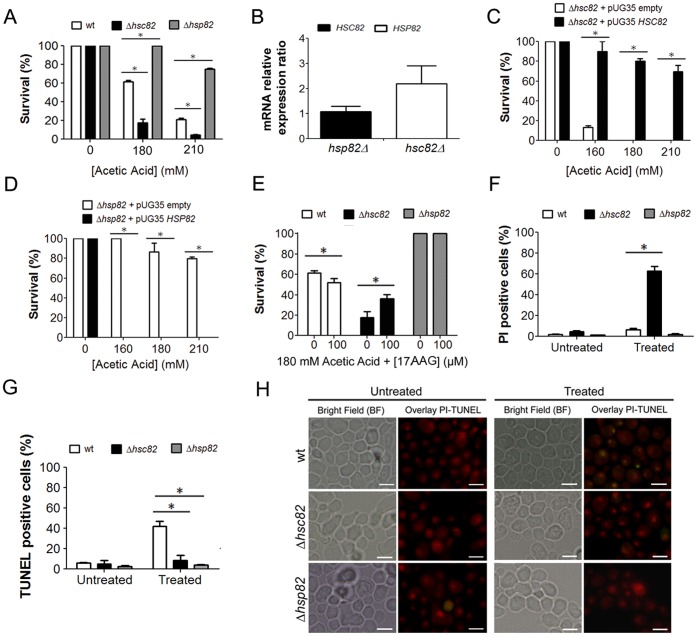
HSP90 isoforms display distinct and antagonistic functions in response to acetic acid treatment. (A) Comparison of the survival rates of wild-type, Δ*hsc82* and Δ*hsp82 Saccharomyces cerevisiae* cells after acetic acid treatment (white bars correspond to wild-type cells, black bars to Δ*hsc82* cells and grey bars to Δ*hsp82* cells; *p<0.05 versus wild-type cells, two-way ANOVA, n = 3). (B) Ratio of *HSC82* and *HSP82* mRNA relative basal expression observed in Δ*hsc82* and Δ*hsp82 S. cerevisiae* cells, respectively, in comparison with wild-type cells. Analysis of the survival rates of (C) Δ*hsc82* and (D) Δ*hsp82 S. cerevisiae* cells harboring a pUG35 plasmid expressing *HSC82* or *HSP82*, after treatment with 0, 160, 180 or 210 mM acetic acid (white bars correspond to cells harboring the empty plasmid and black bars to cells expressing the *HSC82* or *HSP82* gene; *p<0.05 versus cells harboring the empty plasmid, t-test, n = 3). (E) Comparison of the survival rates of wild-type, Δ*hsc82* and Δ*hsp82 S. cerevisiae* cells treated with 180 mM of acetic acid in the presence of 100 µM 17-allylaminogeldanamycin (17AAG), an inhibitor of HSP90 (white bars correspond to wild-type cells, black bars to Δ*hsc82* cells and grey bars to Δ*hsp82* cells; *p<0.05 versus absence of 17AAG, two-way ANOVA, n = 3). (F) Flow cytometry analysis of propidium iodide (PI) staining of wild-type, Δ*hsc82* and Δ*hsp82 S. cerevisiae* cells after acetic acid treatment (180 mM) (white bars correspond to wild-type cells, black bars to Δ*hsc82* cells and grey bars to Δ*hsp82* cells; *p<0.05 versus wild-type cells, two-way ANOVA, n = 3). (G) Percentage of wild-type, Δ*hsc82* and Δ*hsp82* cells displaying TUNEL positive phenotype (white bars correspond to wild-type cells, black bars to Δ*hsc82* cells and grey bars to Δ*hsp82* cells; *p<0.05 versus wild-type cells, two-way ANOVA, n = 3). (H) Epifluorescence and bright field micrographs of untreated and acetic acid treated (180 mM) wild-type, Δ*hsc82* and Δ*hsp82* cells displaying TUNEL reaction to visualize double-strand DNA breaks. Cells were co-stained with propidium iodide in order to facilitate nuclei visualization. TUNEL-positive cells correspond to cells with yellow nuclei due to the overlay between TUNEL reaction, green, and PI staining, red. Bar, 5 µm.

The data presented above show that Hsc82p and Hsp82p are selectively translated during acetic acid treatment. Since these conditions induce apoptotic cell death in 50% of cells, we wished to determine the contribution of these isoforms to the cell death process. For this purpose, the sensitivity of *HSC82* and *HSP82* mutant cells to acetic acid treatment was determined. Deletion of *HSC82* (Δ*hsc82*) resulted in an increased sensitivity, while the deletion of *HSP82* (Δ*hsp82*) enhanced the resistance of cells to acetic acid treatment when compared to the wild-type strain (Figure 5A). Since deletion of one of the genes is predicted to result in transcriptional up-regulation of the other gene we decided to evaluate *HSP82* and *HSC82* mRNA levels in Δ*hsc82* and Δ*hsp82* cells, respectively. Deletion of *HSC82* resulted in an up-regulation of *HSP82* mRNA levels while deletion of *HSP82* had no major effects on *HSC82* mRNA levels (Figure 5B). Together, these results suggest that Hsc82p and Hsp82p play opposite roles in determining the cellular response to acetic acid treatment. Genetic rescue by ectopic expression of *HSC82* or *HSP82* under a *MET25* promoter in the respective deletion strains resulted in a complete reversion of the phenotype (Figure 5C and 5D). In fact, the expression of Hsc82p was able to confer additional protection, while the expression of Hsp82p increased susceptibility to acetic acid treatment (Figure 5C and 5D). These results point once more to HSP90 isoforms antagonistic roles during acetic acid treatment. To further elucidate functions of the HSP90 isoforms, wild-type, Δ*hsc82* and Δ*hsp82* cells were challenged with acetic acid in the presence of 17-allylaminogeldanamycin (17AAG), a small molecule inhibitor of HSP90 [29]. The inhibition of HSP90 had no effect on cell survival in *HSP82* deleted cells. In contrast, inhibition of HSP90 in wild-type cells resulted in increased acetic acid-induced cell death, and in *HSC82* deleted cells led to a moderated protection (Figure 5E). These data further confirm the opposing roles performed by the HSP90 isoforms in response to acetic acid and suggest a pro-survival role for Hsc82p and a pro-death role for the Hsp82p isoform. The evaluation of the type of PCD occurring in each of the above conditions showed that despite the higher sensitivity to acetic acid treatment exhibited by the *HSC82* deleted cells, almost no TUNEL-positive cells were observed by microscopy analysis, in sharp contrast to the known TUNEL-positive phenotype displayed by wild-type cells (Figure 5G and 5H). Moreover, propidium iodine (PI) staining of *HSC82* deleted cells revealed a high percentage of PI positive cells suggesting the occurrence of a necrotic cell death (Figure 5F). These data support the notion [12], [19] that HSP90 isoforms are key molecules in governing the balance between apoptotic and necrotic cell death when cells are challenged with acetic acid.

## Discussion

The involvement of translation in the control of cell death is emerging as a crucial regulatory mechanism both in mammalian and yeast cells [4], [20], [22], [28]. Since translation is the final stage in the flow of genetic information, regulation at this level leads to a direct and immediate response to changes in physiological conditions, such as cell death. During *S. cerevisiae* acetic acid-induced apoptosis, a general amino acid starvation occurs leading to attenuation of general translation that allows cells to conserve resources for a quick reprogramming of gene expression in response to a physiological stimulus [4]. Accordingly, our results show an early impairment in general translation as reflected by the decreased levels of translation initiation factor eIF4A, and by the Gcn2p kinase-dependent-increase in phosphorylation of eIF2α. This result is unexpected and further studies are required to understand the relevance of eIF4A downregulation at early time points of acetic acid treatment.

Yeast Gcn2p kinase is known to phosphorylate eIF2α at serine 51 in response to amino acids starvation, a condition occurring under acetic acid-induced apoptosis [4]. We demonstrate that deletion of *GCN2* restores translation machinery in acetic acid treated cells, as shown by the restored levels of different translation factors and by the decreased eIF2α phosphorylation. Furthermore, deletion of *GCN2* restored cell survival. Thus, the regulation of translation initiation by Gcn2p appears to be a rate-limiting step in the acetic acid-induced apoptotic program. The mechanism activating Gcn2p is most likely intracellular acidification promoted by acetic acid accompanied by the inhibition of aminoacyl-tRNA synthetases, such as lysyl-tRNA synthetase, as we have previously reported [4]. This appears to be a general response of yeast cells to acidic stress [40].

Although our data show that general translation is inhibited shortly after acetic acid treatment (15 min), the levels of eEF1A, eEF2 and eIF4G (a protein required in yeast for cap-independent translation during nutrient-starvation conditions [35]), are only reduced at later time points (120 min) suggesting that it is unlikely that acetic acid is causing defects in elongation in the first two hours of treatment. These data, together with the previous proteomic analysis showing increased translation of specific proteins after acetic acid treatment [4], suggest that alternative translation mechanism(s) are operating soon after the attenuation of global translation initiation. Microarray analysis of polysome-associated mRNAs during acetic acid treatment showed a decrease in the overall mRNA levels, in accordance with the observed inhibition of translation. However, it also revealed a group of mRNAs with increased association with polysomes at 15 and 30 min of acetic acid treatment. We hypothesized that the proteins encoded by these mRNAs would be involved in the cellular response to acetic acid, or in the activation of the cell death process, since the analysis was performed at early time points of the activation and execution of the cell death program. Functional analysis of the microarray data suggests that the cellular protein metabolic process and cytoplasm and organelle organization at 15 min of acetic acid treatment, together with the translation regulation and ribosome structure both at 15 and 30 min of acetic acid treatment were the main functional groups whose mRNAs had increased association with polysomes in response to acetic acid treatment. These data indicate that a remodelling of the cellular content and translation process takes place in response to acetic acid treatment. Notably, two HSP90 isoforms, *HSC82* and *HSP82*, were found to be enriched in the polysome mRNAs, with *HSP82* showing a higher fold increase than *HSC82* after 30 min of acetic acid treatment. Although the molecular mechanism supporting selective translation of these isoforms during acetic acid stress awaits further investigation, mammalian HSP70 and *Drosophila* HSP90 chaperones were shown to be translated by a cap-independent mechanism which utilizes internal ribosome entry sites (IRES) in their respective 5′ UTRs [41], [42]. This offers a possibility that a similar RNA element may be responsible for the selective translation of HSP90 isoforms in yeast. Our data further suggest that the translational control of the HSP90 isoforms is relevant for acetic acid-induced cell death. These data suggest that HSP90 isoforms regulate the balance between apoptosis and necrosis, in accordance with the previously attributed function of HSP90 in the modulation of yeast necrotic cell death [12], [19]. The dual role of HSP90 chaperones may be related to their differential involvement in the folding/refolding of target proteins playing distinct roles in cell death. In fact, although the two isoforms have high homology there are some differencesin amino acid sequence in the N-terminal ATPase domain which may result in distinct protein interactions and therefore divergent roles in response to acetic acid treatment. Of note, HSP90 chaperones are molecular targets for cancer therapy in humans [8], [43]–[49]. The fact that in yeast the two known isoforms of HSP90 chaperones, Hsc82p and Hsp82p, are involved in the modulation of cell death, and given the high conservation of HSP90 chaperones from yeast to mammals, evaluation of the distinct roles of each mammalian HSP90 isoform in the modulation of cell death will be critical to the success of anti-cancer therapies.

## Supporting Information

Figure S1 **Validation of microarray analysis by qPCR of selected genes.** Microarray and qPCR data are shown for mRNAs with increased polysomal association (*CAF20* and *TIF34*) and mRNAs with decreased polysomal association (*CIT2* and *COX3*) upon 15 or 30 min of acetic acid treatment (black bars correspond to microarray analysis and white bars to qPCR data). The values are normalized for untreated control cells and are the mean of triplicate microarray and qPCR determinations.
(TIF)

Figure S2 **qPCR analysis of HSP90 chaperones mRNA levels upon acetic acid treatment.** (A) Illustration of the polysomal fractions from which mRNAs were extracted and analysed by qPCR (M, monosomal fraction; P1, P2, P3 and P4, polysomal fractions). Analysis of the percentage of (B) *HSC82* and (C) *HSP82* mRNA absolute expression levels by qPCR in each of the polysomal fractions of untreated and acetic acid treated cells (195 mM) during 15 or 30 min (white bars correspond to 0 min, black bars correspond to 15 min and grey bars correspond to 30 min of acetic acid treatment).
(TIF)

Table S1 **Microarrays analysis of mRNAs with increased association with polysome fraction upon 15 min of acetic acid treatment.**
(DOC)

Table S2 **Microarrays analysis of mRNAs with increased association with polysome fraction upon 30 min of acetic acid treatment.**
(DOC)
