# Supplementary figures and images for: Involvement of Yeast HSP90 Isoforms in Response to Stress and Cell Death Induced by Acetic Acid
Source: PLoS One. 2013 Aug 15;8(8):e71294. doi: 10.1371/journal.pone.0071294 (PMC3744546; doi:10.1371/journal.pone.0071294)

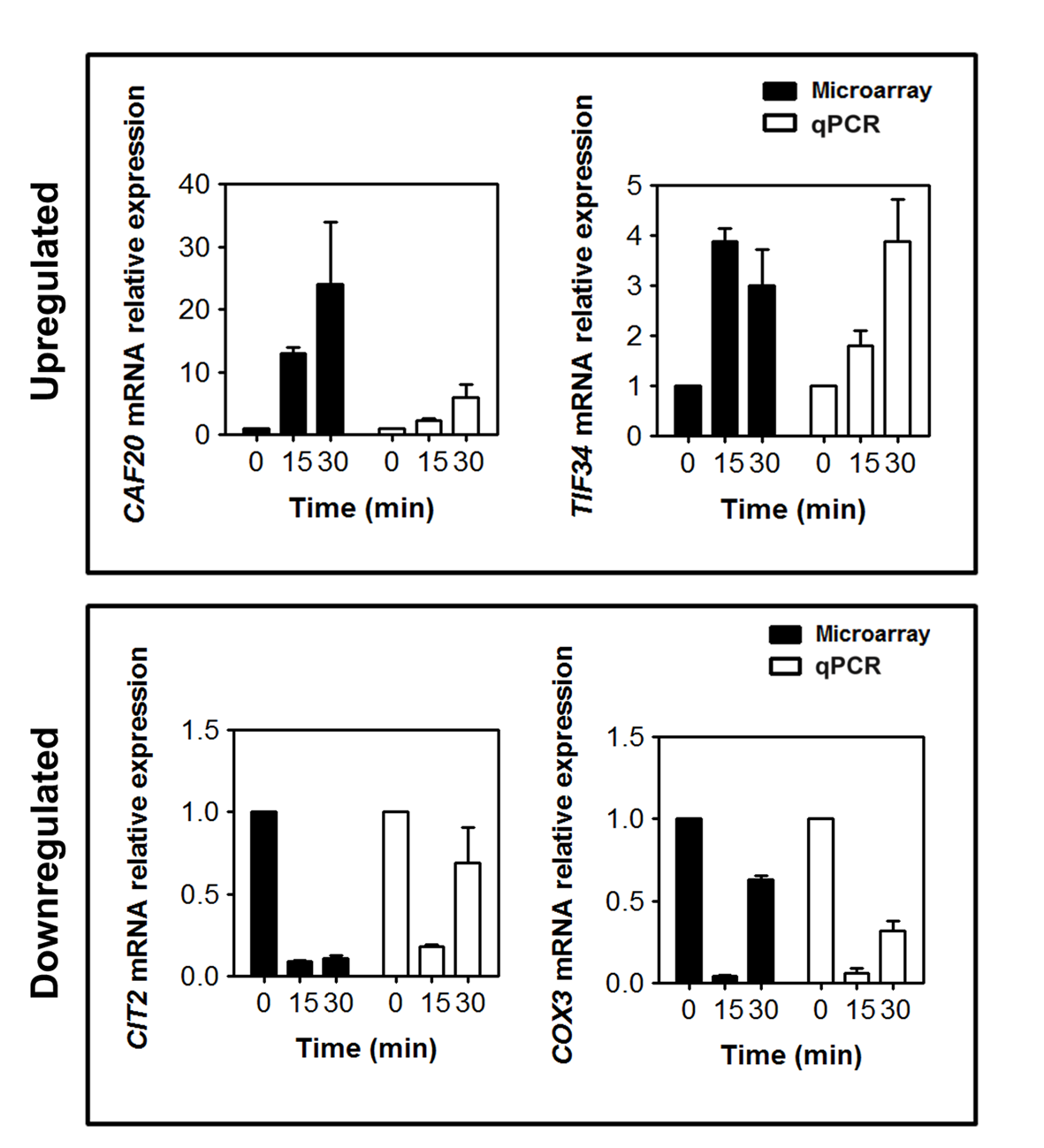

Supplement: Figure S1 — Validation of microarray analysis by qPCR of selected genes. Microarray and qPCR data are shown for mRNAs with increased polysomal association (CAF20 and TIF34) and mRNAs with decreased polysomal association (CIT2 and COX3) upon 15 or 30 min of acetic acid treatment (black bars correspond to microarray analysis and white bars to qPCR data). The values are normalized for untreated control cells and are the mean of triplicate microarray and qPCR determinations. (TIF) [file pone.0071294.s001.tif]

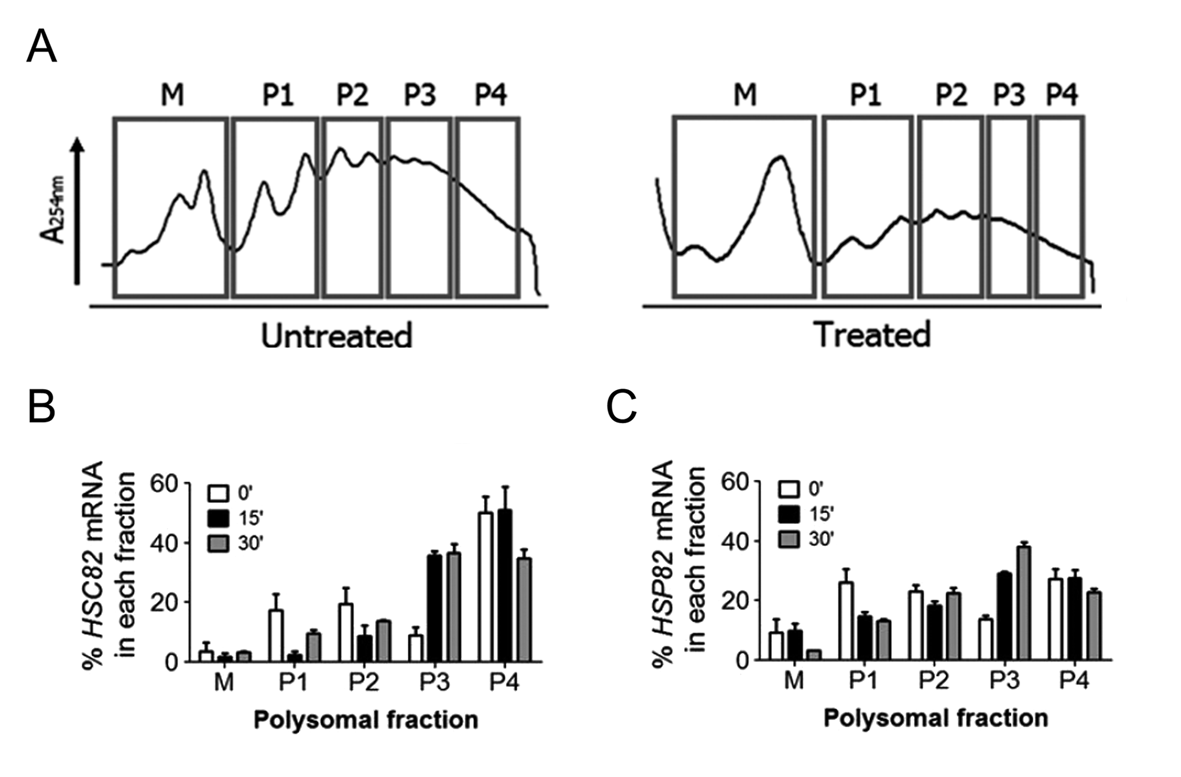

Supplement: Figure S2 — qPCR analysis of HSP90 chaperones mRNA levels upon acetic acid treatment. (A) Illustration of the polysomal fractions from which mRNAs were extracted and analysed by qPCR (M, monosomal fraction; P1, P2, P3 and P4, polysomal fractions). Analysis of the percentage of (B) HSC82 and (C) HSP82 mRNA absolute expression levels by qPCR in each of the polysomal fractions of untreated and acetic acid treated cells (195 mM) during 15 or 30 min (white bars correspond to 0 min, black bars correspond to 15 min and grey bars correspond to 30 min of acetic acid treatment). (TIF) [file pone.0071294.s002.tif]
