# Supplementary material for: Involvement of Yeast HSP90 Isoforms in Response to Stress and Cell Death Induced by Acetic Acid
Source: PLoS One. 2013 Aug 15;8(8):e71294. doi: 10.1371/journal.pone.0071294 (PMC3744546; doi:10.1371/journal.pone.0071294)
Supplement: Table S1 — Microarrays analysis of mRNAs with increased association with polysome fraction upon 15 min of acetic acid treatment. (DOC) [file pone.0071294.s003.doc]

**Table S1.** Microarrays analysis of mRNAs with increased association to polysome fraction upon 15 min of acetic acid treatment.

| **Probe ID** | **Gene symbol** | **Fold change** | ***p*-value** |
| --- | --- | --- | --- |
| YLR027C | *AAT2* | 2.63 | 1.27E-03 |
| YGR037C | *ACB1* | 2.72 | 4.03E-03 |
| YKL192C | *ACP1* | 3.18 | 5.30E-05 |
| YOR128C | *ADE2* | 1.87 | 2.11E-06 |
| YDR408C | *ADE8* | 1.81 | 8.64E-03 |
| YDR226W | *ADK1* | 3.87 | 2.62E-04 |
| YDR214W | *AHA1* | 2.06 | 5.44E-03 |
| YJR047C | *ANB1* | 5.12 | 8.29E-04 |
| YPR128C | *ANT1* | 2.26 | 7.26E-05 |
| YLR102C | *APC9* | 2.27 | 9.67E-03 |
| YML022W | *APT1* | 2.87 | 3.78E-03 |
| YBR149W | *ARA1* | 2.42 | 1.49E-03 |
| YGL105W | *ARC1* | 2.93 | 5.49E-03 |
| YIL062C | *ARC15* | 3.01 | 9.90E-04 |
| YKL013C | *ARC19* | 2.21 | 2.38E-03 |
| YHR013C | *ARD1* | 4.79 | 1.96E-04 |
| YDL100C | *ARR4* | 2.46 | 3.97E-03 |
| YGL017W | *ATE1* | 1.86 | 3.14E-07 |
| YNL315C | *ATP11* | 2.29 | 2.14E-06 |
| YDR298C | *ATP5* | 1.79 | 1.60E-04 |
| YKL016C | *ATP7* | 2.65 | 1.57E-04 |
| YFL010W-A | *AUA1* | 3.52 | 1.31E-03 |
| YER155C | *BEM2* | 1.43 | 5.06E-03 |
| YIL004C | *BET1* | 1.92 | 4.76E-03 |
| YPR176C | *BET2* | 2.35 | 1.33E-04 |
| YJL031C | *BET4* | 4.84 | 9.39E-04 |
| YFR047C | *BNA6* | 4.46 | 1.72E-04 |
| YLR078C | *BOS1* | 2.56 | 2.55E-03 |
| YHR114W | *BZZ1* | 2.44 | 7.20E-04 |
| YOR276W | *CAF20* | 6.02 | 3.49E-06 |
| YNL288W | *CAF40* | 2.32 | 9.24E-05 |
| YER048C | *CAJ1* | 2.75 | 5.77E-05 |
| YOR125C | *CAT5* | 2.41 | 1.97E-04 |
| YPL178W | *CBC2* | 2.95 | 4.30E-04 |
| YPL215W | *CBP3* | 2.26 | 4.44E-06 |
| YIL142W | *CCT2* | 1.73 | 6.42E-06 |
| YDL143W | *CCT4* | 3.12 | 1.75E-04 |
| YCR002C | *CDC10* | 2.24 | 1.11E-03 |
| YDL165W | *CDC36* | 1.74 | 1.73E-03 |
| YDR168W | *CDC37* | 2.21 | 8.91E-04 |
| YDL126C | *CDC48* | 2.64 | 1.80E-03 |
| YLR418C | *CDC73* | 2.67 | 5.06E-05 |
| YJR057W | *CDC8* | 2.13 | 4.20E-03 |
| YBR109C | *CMD1* | 2.15 | 6.21E-05 |
| YLL009C | *COX17* | 5.46 | 1.27E-05 |
| YIL111W | *COX5B* | 3.61 | 1.03E-06 |
| YML078W | *CPR3* | 4.73 | 1.11E-04 |
| YLR216C | *CPR6* | 2.82 | 3.81E-03 |
| YNL027W | *CRZ1* | 2.01 | 4.21E-04 |
| YHR191C | *CTF8* | 2.50 | 2.84E-04 |
| YOR042W | *CUE5* | 3.47 | 9.22E-05 |
| YGL166W | *CUP2* | 1.68 | 6.81E-06 |
| YDR163W | *CWC15* | 3.03 | 1.69E-03 |
| YKL096W | *CWP1* | 5.52 | 3.12E-04 |
| YAL039C | *CYC3* | 2.26 | 6.19E-03 |
| YAL012W | *CYS3* | 3.64 | 1.54E-04 |
| YGR155W | *CYS4* | 2.77 | 1.81E-03 |
| YML113W | *DAT1* | 1.42 | 5.27E-03 |
| YOR046C | *DBP5* | 3.55 | 2.58E-05 |
| YOL149W | *DCP1* | 3.13 | 6.21E-06 |
| YOR163W | *DDP1* | 3.18 | 8.42E-04 |
| YKL054C | *DEF1* | 2.42 | 2.03E-03 |
| YIR004W | *DJP1* | 1.73 | 1.28E-06 |
| YDR121W | *DPB4* | 2.37 | 1.39E-03 |
| YMR276W | *DSK2* | 4.41 | 2.22E-06 |
| YDR424C | *DYN2* | 2.81 | 2.04E-04 |
| YLR390W | *ECM19* | 4.29 | 1.21E-03 |
| YHL030W | *ECM29* | 3.32 | 4.05E-04 |
| YBL047C | *EDE1* | 2.48 | 5.94E-03 |
| YHR193C | *EGD2* | 3.56 | 9.13E-03 |
| YNL084C | *END3* | 3.19 | 3.10E-04 |
| YJR125C | *ENT3* | 5.05 | 1.33E-05 |
| YDR153C | *ENT5* | 2.17 | 1.67E-03 |
| YPL028W | *ERG10* | 3.24 | 3.37E-03 |
| YML126C | *ERG13* | 3.00 | 7.82E-03 |
| YJL167W | *ERG20* | 3.21 | 7.46E-05 |
| YGL001C | *ERG26* | 3.34 | 9.03E-05 |
| YLR100W | *ERG27* | 2.97 | 1.25E-04 |
| YHR190W | *ERG9* | 2.34 | 4.07E-03 |
| YDL166C | *FAP7* | 3.00 | 5.90E-03 |
| YPL231W | *FAS2* | 1.64 | 2.32E-03 |
| YPR062W | *FCY1* | 2.46 | 7.16E-03 |
| YIL065C | *FIS1* | 2.63 | 2.87E-03 |
| YIL098C | *FMC1* | 2.09 | 3.99E-04 |
| YNL135C | *FPR1* | 3.37 | 4.88E-05 |
| YML074C | *FPR3* | 2.74 | 6.42E-04 |
| YFL022C | *FRS2* | 2.10 | 7.54E-03 |
| YHR049W | *FSH1* | 4.94 | 1.52E-04 |
| YMR222C | *FSH2* | 3.70 | 1.33E-03 |
| YAL035W | *FUN12* | 2.55 | 2.14E-04 |
| YER027C | *GAL83* | 2.01 | 5.72E-03 |
| YCL011C | *GBP2* | 1.68 | 6.76E-03 |
| YGR083C | *GCD2* | 2.86 | 6.52E-08 |
| YFR009W | *GCN20* | 2.50 | 3.48E-03 |
| YAL044C | *GCV3* | 2.93 | 3.96E-04 |
| YLR094C | *GIS3* | 2.00 | 2.38E-03 |
| YOR168W | *GLN4* | 6.04 | 3.46E-04 |
| YML004C | *GLO1* | 3.93 | 1.81E-03 |
| YDR272W | *GLO2* | 5.92 | 1.07E-04 |
| YHL031C | *GOS1* | 2.47 | 5.79E-03 |
| YLR293C | *GSP1* | 2.27 | 7.36E-03 |
| YGL181W | *GTS1* | 2.21 | 5.20E-04 |
| YIR038C | *GTT1* | 3.55 | 5.01E-04 |
| YNL281W | *HCH1* | 2.30 | 1.28E-06 |
| YLR192C | *HCR1* | 1.85 | 1.06E-03 |
| YOR176W | *HEM15* | 5.04 | 3.09E-08 |
| YBR248C | *HIS7* | 2.32 | 8.05E-03 |
| YDL125C | *HNT1* | 5.89 | 4.54E-04 |
| YDR305C | *HNT2* | 2.76 | 2.52E-06 |
| YJR139C | *HOM6* | 2.99 | 1.46E-03 |
| YEL066W | *HPA3* | 2.76 | 3.01E-04 |
| YMR186W | *HSC82* | 2.72 | 5.86E-03 |
| YHL002W | *HSE1* | 3.49 | 5.51E-03 |
| YOR020C | *HSP10* | 3.25 | 4.77E-03 |
| YLR259C | *HSP60* | 2.51 | 3.01E-03 |
| YIR037W | *HYR1* | 5.35 | 8.62E-08 |
| YJR016C | *ILV3* | 2.48 | 3.29E-03 |
| YCR046C | *IMG1* | 2.95 | 5.51E-03 |
| YBR011C | *IPP1* | 2.55 | 2.77E-07 |
| YNL265C | *IST1* | 3.32 | 2.55E-03 |
| YBR245C | *ISW1* | 1.59 | 1.86E-03 |
| YPR133C | *IWS1* | 2.95 | 5.75E-04 |
| YGL173C | *KEM1* | 2.36 | 5.06E-04 |
| YDR148C | *KGD2* | 1.67 | 2.54E-03 |
| YKL183W | *LOT5* | 3.29 | 1.89E-03 |
| YBL026W | *LSM2* | 3.70 | 2.66E-03 |
| YER112W | *LSM4* | 1.83 | 2.44E-03 |
| YPL004C | *LSP1* | 3.81 | 4.17E-03 |
| YMR038C | *LYS7* | 3.01 | 3.75E-04 |
| YGL086W | *MAD1* | 3.23 | 2.86E-03 |
| YIL070C | *MAM33* | 6.55 | 2.58E-04 |
| YBL091C | *MAP2* | 2.87 | 4.33E-03 |
| YOR197W | *MCA1* | 3.58 | 2.24E-04 |
| YDL078C | *MDH3* | 6.34 | 5.33E-04 |
| YLR303W | *MET17* | 2.44 | 3.06E-03 |
| YOR241W | *MET7* | 1.63 | 5.78E-03 |
| YOR232W | *MGE1* | 3.44 | 6.51E-03 |
| YKR095W | *MLP1* | 1.45 | 3.00E-03 |
| YIL051C | *MMF1* | 3.26 | 7.05E-05 |
| YJR074W | *MOG1* | 2.82 | 1.43E-04 |
| YDR347W | *MRP1* | 3.93 | 2.87E-03 |
| YKL003C | *MRP17* | 1.77 | 1.73E-03 |
| YBL038W | *MRPL16* | 2.40 | 1.83E-03 |
| YKR085C | *MRPL20* | 4.25 | 2.04E-03 |
| YOR150W | *MRPL23* | 2.27 | 3.53E-03 |
| YBR282W | *MRPL27* | 2.59 | 9.64E-04 |
| YDR462W | *MRPL28* | 2.27 | 6.69E-03 |
| YKL138C | *MRPL31* | 1.97 | 6.68E-03 |
| YJL096W | *MRPL49* | 5.12 | 4.39E-04 |
| YPR100W | *MRPL51* | 2.16 | 6.48E-04 |
| YJL063C | *MRPL8* | 3.00 | 1.13E-04 |
| YGR220C | *MRPL9* | 2.30 | 5.45E-03 |
| YBR251W | *MRPS5* | 1.92 | 4.74E-04 |
| YMR158W | *MRPS8* | 3.95 | 1.07E-03 |
| YOR354C | *MSC6* | 1.39 | 8.35E-03 |
| YGL122C | *NAB2* | 2.12 | 1.78E-03 |
| YGR232W | *NAS6* | 3.63 | 1.38E-04 |
| YDL040C | *NAT1* | 2.23 | 1.60E-03 |
| YPR052C | *NHP6A* | 2.29 | 3.51E-03 |
| YBR089C-A | *NHP6B* | 2.99 | 7.69E-8 |
| YGL221C | *NIF3* | 2.16 | 8.73E-03 |
| YPL211W | *NIP7* | 2.34 | 3.68E-03 |
| YLR351C | *NIT3* | 3.16 | 2.26E-04 |
| YOL041C | *NOP12* | 2.20 | 2.14E-03 |
| YNL175C | *NOP13* | 4.61 | 2.44E-06 |
| YPR072W | *NOT5* | 4.42 | 3.14E-04 |
| YMR091C | *NPL6* | 1.76 | 2.63E-03 |
| YER126C | *NSA2* | 2.30 | 5.19E-06 |
| YER009W | *NTF2* | 3.86 | 2.01E-03 |
| YER006W | *NUG1* | 3.11 | 2.72E-06 |
| YIL115C | *NUP159* | 2.26 | 1.98E-03 |
| YBR129C | *OPY1* | 1.94 | 3.88E-03 |
| YHR063C | *PAN5* | 2.19 | 5.26E-05 |
| YDR228C | *PCF11* | 1.60 | 3.89E-03 |
| YNL231C | *PDR16* | 1.79 | 1.74E-06 |
| YER153C | *PET122* | 3.80 | 1.71E-03 |
| YOR158W | *PET123* | 1.54 | 3.02E-03 |
| YOL044W | *PEX15* | 4.07 | 1.59E-04 |
| YPL112C | *PEX25* | 2.61 | 4.14E-03 |
| YOR122C | *PFY1* | 2.83 | 4.22E-04 |
| YGL025C | *PGD1* | 2.40 | 8.95E-03 |
| YPL031C | *PHO85* | 3.07 | 4.32E-03 |
| YNL055C | *POR1* | 3.25 | 8.57E-04 |
| YDL134C | *PPH21* | 1.99 | 2.40E-03 |
| YDL188C | *PPH22* | 2.87 | 6.01E-03 |
| YMR314W | *PRE5* | 2.45 | 2.26E-04 |
| YOR323C | *PRO2* | 3.16 | 4.17E-04 |
| YBL068W | *PRS4* | 3.24 | 7.72E-05 |
| YJL166W | *QCR8* | 1.46 | 6.71E-03 |
| YDL103C | *QRI1* | 3.57 | 2.00E-03 |
| YMR022W | *QRI8* | 2.39 | 3.89E-04 |
| YER095W | *RAD51* | 1.68 | 3.06E-03 |
| YNL216W | *RAP1* | 1.88 | 4.46E-06 |
| YCR036W | *RBK1* | 2.18 | 2.69E-04 |
| YLR248W | *RCK2* | 3.24 | 2.12E-04 |
| YJL204C | *RCY1* | 1.67 | 2.97E-03 |
| YNL312W | *RFA2* | 2.22 | 2.53E-03 |
| YJL173C | *RFA3* | 1.82 | 8.74E-03 |
| YDR487C | *RIB3* | 2.80 | 2.97E-04 |
| YER083C | *RMD7* | 3.56 | 3.26E-04 |
| YCL028W | *RNQ1* | 4.78 | 1.63E-05 |
| YDR156W | *RPA14* | 2.23 | 1.90E-04 |
| YOL005C | *RPB11* | 3.30 | 7.65E-04 |
| YJL140W | *RPB4* | 4.93 | 3.93E-03 |
| YDR404C | *RPB7* | 1.39 | 8.89E-03 |
| YJL121C | *RPE1* | 4.23 | 1.03E-06 |
| YMR121C | *RPL15B* | 3.33 | 4.34E-06 |
| YNL069C | *RPL16B* | 4.10 | 5.61E-03 |
| YPL079W | *RPL21B* | 4.02 | 1.50E-03 |
| YBL087C | *RPL23A* | 2.05 | 2.01E-03 |
| YDR471W | *RPL27B* | 3.54 | 2.94E-04 |
| YBR031W | *RPL4A* | 4.19 | 5.85E-03 |
| YDR012W | *RPL4B* | 3.28 | 2.90E-03 |
| YLL045C | *RPL8B* | 2.61 | 6.70E-03 |
| YDL097C | *RPN6* | 3.02 | 4.64E-03 |
| YPR187W | *RPO26* | 5.19 | 4.23E-03 |
| YDR064W | *RPS13* | 3.57 | 1.84E-03 |
| YCR031C | *RPS14A* | 2.17 | 2.68E-06 |
| YJL191W | *RPS14B* | 2.45 | 1.99E-03 |
| YOL121C | *RPS19A* | 3.61 | 9.20E-03 |
| YJL190C | *RPS22A* | 5.26 | 6.38E-03 |
| YLR367W | *RPS22B* | 2.91 | 1.35E-03 |
| YKL156W | *RPS27A* | 2.04 | 9.22E-03 |
| YLR167W | *RPS31* | 5.43 | 6.48E-03 |
| YPL090C | *RPS6A* | 3.67 | 7.29E-03 |
| YKL145W | *RPT1* | 2.80 | 4.85E-04 |
| YMR131C | *RRB1* | 2.27 | 9.35E-03 |
| YDL111C | *RRP42* | 1.59 | 5.89E-03 |
| YER050C | *RSM18* | 3.00 | 1.48E-03 |
| YJR113C | *RSM7* | 3.90 | 3.85E-03 |
| YGL244W | *RTF1* | 1.83 | 2.59E-03 |
| YOR216C | *RUD3* | 3.51 | 2.02E-03 |
| YPL235W | *RVB2* | 2.21 | 6.73E-03 |
| YCR009C | *RVS161* | 2.80 | 2.84E-05 |
| YDR388W | *RVS167* | 2.66 | 3.95E-04 |
| YLR180W | *SAM1* | 2.83 | 9.18E-04 |
| YMR263W | *SAP30* | 3.16 | 2.72E-04 |
| YPL218W | *SAR1* | 4.35 | 1.40E-03 |
| YHL034C | *SBP1* | 3.19 | 9.23E-03 |
| YGL011C | *SCL1* | 2.20 | 1.05E-03 |
| YJL080C | *SCP160* | 3.62 | 1.87E-03 |
| YER120W | *SCS2* | 4.53 | 5.15E-04 |
| YPL085W | *SEC16* | 1.79 | 2.49E-04 |
| YBL050W | *SEC17* | 2.83 | 2.29E-04 |
| YLR268W | *SEC22* | 3.15 | 2.99E-03 |
| YDR170C | *SEC7* | 2.77 | 7.48E-07 |
| YLR292C | *SEC72* | 2.73 | 7.77E-04 |
| YGR009C | *SEC9* | 2.84 | 2.44E-03 |
| YIL074C | *SER33* | 1.80 | 7.77E-03 |
| YDL168W | *SFA1* | 1.95 | 5.51E-04 |
| YJL145W | *SFH5* | 3.22 | 2.67E-04 |
| YJR134C | *SGM1* | 1.91 | 3.53E-03 |
| YKL130C | *SHE2* | 2.04 | 8.07E-03 |
| YBR258C | *SHG1* | 3.59 | 7.61E-05 |
| YBL058W | *SHP1* | 3.18 | 4.82E-03 |
| YNL236W | *SIN4* | 2.88 | 4.57E-03 |
| YGL208W | *SIP2* | 2.16 | 7.16E-03 |
| YDR328C | *SKP1* | 2.64 | 3.24E-03 |
| YML058W | *SML1* | 1.96 | 9.67E-03 |
| YFL017W-A | *SMX2* | 4.72 | 5.12E-04 |
| YPR182W | *SMX3* | 5.10 | 3.49E-03 |
| YKL079W | *SMY1* | 3.44 | 6.56E-04 |
| YAL030W | *SNC1* | 4.01 | 1.46E-03 |
| YGL115W | *SNF4* | 4.64 | 1.56E-06 |
| YJR104C | *SOD1* | 5.51 | 8.67E-05 |
| YHR163W | *SOL3* | 2.07 | 4.89E-04 |
| YGL093W | *SPC105* | 2.84 | 5.74E-04 |
| YER018C | *SPC25* | 2.29 | 6.38E-03 |
| YPR069C | *SPE3* | 3.14 | 3.49E-06 |
| YGL207W | *SPT16* | 1.74 | 9.49E-06 |
| YDR292C | *SRP101* | 2.05 | 8.60E-03 |
| YOR027W | *STI1* | 4.12 | 5.54E-04 |
| YLR150W | *STM1* | 3.32 | 4.83E-03 |
| YPL237W | *SUI3* | 4.08 | 1.74E-03 |
| YDR172W | *SUP35* | 3.03 | 9.06E-05 |
| YDR167W | *TAF10* | 2.04 | 1.02E-03 |
| YJL052W | *TDH1* | 1.64 | 4.01E-03 |
| YHR025W | *THR1* | 1.73 | 9.45E-03 |
| YMR146C | *TIF34* | 2.57 | 2.61E-03 |
| YGL049C | *TIF4632* | 1.82 | 1.53E-04 |
| YJL143W | *TIM17* | 2.28 | 1.04E-03 |
| YJR135W-A | *TIM8* | 3.72 | 2.67E-04 |
| YEL020W-A | *TIM9* | 4.50 | 3.58E-06 |
| YER011W | *TIR1* | 2.27 | 4.51E-04 |
| YIL011W | *TIR3* | 1.91 | 3.96E-03 |
| YNL070W | *TOM7* | 3.08 | 2.72E-03 |
| YAL016W | *TPD3* | 2.92 | 2.96E-03 |
| YDR353W | *TRR1* | 1.96 | 1.18E-03 |
| YOR115C | *TRS33* | 3.01 | 7.10E-03 |
| YGR209C | *TRX2* | 4.38 | 5.92E-04 |
| YCR083W | *TRX3* | 2.84 | 3.46E-04 |
| YML028W | *TSA1* | 3.58 | 1.42E-05 |
| YBR058C-A | *TSC3* | 3.44 | 2.36E-03 |
| YDR513W | *TTR1* | 3.05 | 8.01E-03 |
| YER100W | *UBC6* | 2.46 | 1.69E-04 |
| YLL039C | *UBI4* | 3.68 | 6.60E-03 |
| YFR010W | *UBP6* | 2.65 | 5.26E-03 |
| YKL024C | *URA6* | 2.55 | 5.45E-04 |
| YDR400W | *URH1* | 2.39 | 2.09E-03 |
| YDL058W | *USO1* | 2.52 | 5.15E-04 |
| YGR128C | *UTP8* | 1.85 | 4.91E-03 |
| YEL038W | *UTR4* | 1.79 | 4.15E-03 |
| YHR039C-A | *VMA10* | 7.89 | 3.15E-03 |
| YPR036W | *VMA13* | 2.92 | 2.40E-03 |
| YOR132W | *VPS17* | 2.36 | 8.94E-06 |
| YKL041W | *VPS24* | 1.31 | 6.31E-03 |
| YFL010C | *WWM1* | 3.36 | 9.91E-06 |
| YPL252C | *YAH1* | 2.68 | 5.79E-04 |
| YHR135C | *YCK1* | 1.98 | 7.08E-03 |
| YDL120W | *YFH1* | 1.96 | 2.08E-04 |
| YDL198C | *YHM1* | 5.25 | 8.22E-05 |
| YNL263C | *YIF1* | 2.10 | 9.55E-04 |
| YNL044W | *YIP3* | 2.33 | 5.80E-03 |
| YLR200W | *YKE2* | 1.63 | 2.08E-03 |
| YML025C | *YML6* | 1.74 | 7.93E-03 |
| YKL067W | *YNK1* | 2.59 | 3.91E-04 |
| YPR028W | *YOP1* | 2.44 | 5.75E-04 |
| YML027W | *YOX1* | 1.92 | 6.88E-03 |
| YDL235C | *YPD1* | 2.35 | 8.33E-03 |
| YDR368W | *YPR1* | 1.84 | 2.13E-03 |
| YFL038C | *YPT1* | 2.34 | 1.65E-03 |
| YML001W | *YPT7* | 4.53 | 2.95E-05 |
| YIL063C | *YRB2* | 2.30 | 9.77E-03 |
