# Supplementary material for: Involvement of Yeast HSP90 Isoforms in Response to Stress and Cell Death Induced by Acetic Acid
Source: PLoS One. 2013 Aug 15;8(8):e71294. doi: 10.1371/journal.pone.0071294 (PMC3744546; doi:10.1371/journal.pone.0071294)
Supplement: Table S2 — Microarrays analysis of mRNAs with increased association with polysome fraction upon 30 min of acetic acid treatment. (DOC) [file pone.0071294.s004.doc]

**Table S2.** Microarrays analysis of mRNAs with increased association to polysome fraction upon 30 min of acetic acid treatment.

| **Probe ID** | **Gene Symbol** | **Fold change** | ***p*-value** |
| --- | --- | --- | --- |
| YKL192C | *ACP1* | 2.83 | 2.38E-03 |
| YDR226W | *ADK1* | 3.19 | 1.02E-02 |
| YBR059C | *AKL1* | 2.69 | 2.52E-02 |
| YOR374W | *ALD4* | 2.73 | 3.01E-02 |
| YJR047C | *ANB1* | 3.46 | 2.26E-02 |
| YIL062C | *ARC15* | 4.14 | 7.00E-02 |
| YKL185W | *ASH1* | 4.77 | 4.18E-02 |
| YGR097W | *ASK10* | 1.93 | 2.22E-05 |
| YKL016C | *ATP7* | 3.07 | 6.02E-02 |
| YER155C | *BEM2* | 1.59 | 7.74E-02 |
| YPR176C | *BET2* | 1.53 | 4.39E-02 |
| YJL060W | *BNA3* | 2.33 | 8.40E-02 |
| YOR276W | *CAF20* | 6.56 | 3.49E-02 |
| YNL288W | *CAF40* | 2.01 | 1.18E-02 |
| YER048C | *CAJ1* | 2.01 | 8.08E-02 |
| YDL126C | *CDC48* | 2.80 | 2.76E-02 |
| YER164W | *CHD1* | 2.10 | 1.51E-06 |
| YLL009C | *COX17* | 4.07 | 4.60E-02 |
| YML078W | *CPR3* | 4.43 | 1.22E-02 |
| YOR042W | *CUE5* | 2.63 | 1.58E-03 |
| YAL012W | *CYS3* | 3.06 | 5.02E-03 |
| YGR155W | *CYS4* | 2.47 | 9.78E-03 |
| YKL054C | *DEF1* | 2.36 | 8.46E-02 |
| YBR201W | *DER1* | 1.83 | 5.48E-03 |
| YLL001W | *DNM1* | 1.67 | 5.88E-02 |
| YNR067C | *DSE4* | 2.23 | 3.02E-02 |
| YKR076W | *ECM4* | 3.07 | 6.20E-03 |
| YNL327W | *EGT2* | 1.98 | 6.72E-02 |
| YGR175C | *ERG1* | 2.01 | 8.72E-02 |
| YML126C | *ERG13* | 3.52 | 1.16E-02 |
| YLR100W | *ERG27* | 2.58 | 3.27E-02 |
| YOR280C | *FSH3* | 1.75 | 1.64E-02 |
| YLR094C | *GIS3* | 2.71 | 5.98E-02 |
| YMR311C | *GLC8* | 2.05 | 1.56E-02 |
| YOR168W | *GLN4* | 3.64 | 9.80E-03 |
| YPR160W | *GPH1* | 4.00 | 7.57E-02 |
| YLR293C | *GSP1* | 2.10 | 2.40E-02 |
| YDL223C | *HBT1* | 3.50 | 9.13E-03 |
| YOR176W | *HEM15* | 4.48 | 1.83E-02 |
| YML075C | *HMG1* | 1.40 | 9.45E-02 |
| YDL125C | *HNT1* | 5.18 | 1.43E-02 |
| YJR139C | *HOM6* | 2.87 | 6.67E-02 |
| YMR186W | *HSC82* | 2.81 | 3.68E-03 |
| YFL014W | *HSP12* | 2.41 | 1.13E-02 |
| YLR259C | *HSP60* | 2.62 | 2.30E-02 |
| YPL240C | *HSP82* | 3.20 | 2.08E-02 |
| YJR016C | *ILV3* | 2.42 | 7.55E-02 |
| YCL009C | *ILV6* | 2.37 | 8.14E-03 |
| YGL173C | *KEM1* | 1.71 | 4.73E-02 |
| YNL132W | *KRE33* | 1.68 | 2.69E-02 |
| YIL070C | *MAM33* | 2.56 | 6.88E-02 |
| YBL091C | *MAP2* | 3.12 | 9.12E-02 |
| YGL209W | *MIG2* | 2.87 | 9.12E-02 |
| YPL082C | *MOT1* | 3.34 | 9.15E-05 |
| YPR166C | *MRP2* | 2.01 | 7.13E-07 |
| YKR085C | *MRPL20* | 3.50 | 2.99E-05 |
| YOR150W | *MRPL23* | 2.16 | 4.36E-03 |
| YBR282W | *MRPL27* | 2.75 | 2.4E-05 |
| YJL096W | *MRPL49* | 4.32 | 3.95E-02 |
| YGR220C | *MRPL9* | 2.17 | 7.83E-04 |
| YMR158W | *MRPS8* | 2.65 | 3.64E-02 |
| YOR354C | *MSC6* | 1.47 | 1.61E-02 |
| YMR109W | *MYO5* | 2.69 | 7.07E-04 |
| YNL137C | *NAM9* | 1.99 | 9.98E-02 |
| YGR232W | *NAS6* | 4.10 | 4.04E-02 |
| YDR176W | *NGG1* | 1.23 | 3.23E-02 |
| YPR052C | *NHP6A* | 3.17 | 1.59E-03 |
| YOL041C | *NOP12* | 2.04 | 9.83E-04 |
| YNL175C | *NOP13* | 3.31 | 3.36E-02 |
| YMR091C | *NPL6* | 1.57 | 5.41E-05 |
| YJR042W | *NUP85* | 1.33 | 1.11E-02 |
| YIR006C | *PAN1* | 3.82 | 3.37E-02 |
| YNL015W | *PBI2* | 2.98 | 7.54E-03 |
| YLR134W | *PDC5* | 1.92 | 4.19E-02 |
| YER153C | *PET122* | 2.86 | 4.85E-02 |
| YKL164C | *PIR1* | 1.78 | 2.50E-02 |
| YNL055C | *POR1* | 3.05 | 4.94E-05 |
| YDL188C | *PPH22* | 4.07 | 1.64E-05 |
| YBL068W | *PRS4* | 2.91 | 4.27E-05 |
| YDR055W | *PST1* | 3.44 | 1.27E-02 |
| YOR265W | *RBL2* | 2.15 | 6.48E-02 |
| YCL028W | *RNQ1* | 4.70 | 2.93E-03 |
| YDR156W | *RPA14* | 3.76 | 1.66E-03 |
| YOL005C | *RPB11* | 3.36 | 1.35E-02 |
| YDR418W | *RPL12B* | 2.46 | 1.91E-02 |
| YNL069C | *RPL16B* | 3.11 | 2.98E-03 |
| YBR191W | *RPL21A* | 2.05 | 5.67E-02 |
| YBL087C | *RPL23A* | 1.71 | 8.54E-03 |
| YDR471W | *RPL27B* | 3.57 | 5.24E-03 |
| YFL036W | *RPO41* | 2.21 | 6.43E-03 |
| YDR064W | *RPS13* | 2.75 | 1.46E-02 |
| YJL191W | *RPS14B* | 1.91 | 9.91E-02 |
| YOL040C | *RPS15* | 2.06 | 4.92E-02 |
| YOL121C | *RPS19A* | 2.89 | 1.93E-02 |
| YER074W | *RPS24A* | 2.41 | 1.36E-04 |
| YIL069C | *RPS24B* | 2.62 | 7.16E-04 |
| YGR027C | *RPS25A* | 2.24 | 9.46E-02 |
| YGL189C | *RPS26A* | 3.57 | 1.64E-03 |
| YER131W | *RPS26B* | 2.97 | 8.33E-02 |
| YKL156W | *RPS27A* | 2.23 | 4.07E-02 |
| YDL061C | *RPS29B* | 3.66 | 1.75E-02 |
| YJR145C | *RPS4A* | 2.11 | 1.39E-02 |
| YHR203C | *RPS4B* | 1.81 | 6.49E-02 |
| YPL090C | *RPS6A* | 2.77 | 1.04E-02 |
| YBR181C | *RPS6B* | 3.53 | 4.04E-02 |
| YOR096W | *RPS7A* | 1.93 | 5.38E-02 |
| YDL111C | *RRP42* | 1.89 | 2.85E-02 |
| YMR229C | *RRP5* | 3.53 | 4.89E-02 |
| YDR129C | *SAC6* | 2.55 | 8.61E-02 |
| YFR040W | *SAP155* | 2.20 | 8.94E-03 |
| YMR263W | *SAP30* | 2.45 | 1.30E-02 |
| YER120W | *SCS2* | 3.36 | 5.55E-03 |
| YPL085W | *SEC16* | 2.11 | 4.90E-02 |
| YBR080C | *SEC18* | 1.73 | 1.95E-02 |
| YDR170C | *SEC7* | 2.24 | 7.33E-06 |
| YGR009C | *SEC9* | 2.78 | 1.52E-02 |
| YJL145W | *SFH5* | 2.16 | 2.78E-05 |
| YJR134C | *SGM1* | 2.14 | 4.02E-03 |
| YDR393W | *SHE9* | 2.31 | 6.03E-02 |
| YBR258C | *SHG1* | 3.06 | 3.34E-03 |
| YNL007C | *SIS1* | 2.20 | 1.92E-02 |
| YNL167C | *SKO1* | 2.63 | 2.48E-02 |
| YBL007C | *SLA1* | 3.12 | 8.71E-02 |
| YFL017W-A | *SMX2* | 4.89 | 2.02E-02 |
| YPR182W | *SMX3* | 4.85 | 6.51E-03 |
| YKL079W | *SMY1* | 3.74 | 4.16E-02 |
| YDR525W-A | *SNA2* | 4.32 | 2.26E-02 |
| YAL030W | *SNC1* | 6.55 | 4.60E-02 |
| YJR104C | *SOD1* | 6.13 | 6.05E-02 |
| YGL093W | *SPC105* | 2.14 | 3.27E-02 |
| YHR139C | *SPS100* | 2.29 | 9.52E-05 |
| YGR116W | *SPT6* | 1.62 | 6.94E-02 |
| YBR169C | *SSE2* | 3.26 | 2.72E-02 |
| YOR027W | *STI1* | 3.88 | 3.30E-02 |
| YLR150W | *STM1* | 2.46 | 2.96E-02 |
| YMR054W | *STV1* | 2.15 | 7.40E-02 |
| YDR172W | *SUP35* | 3.07 | 1.30E-03 |
| YPL016W | *SWI1* | 4.40 | 7.28E-03 |
| YJL052W | *TDH1* | 3.06 | 8.20E-02 |
| YGR186W | *TFG1* | 2.19 | 8.67E-03 |
| YGR181W | *TIM13* | 2.33 | 1.35E-05 |
| YER011W | *TIR1* | 1.86 | 5.96E-02 |
| YNL070W | *TOM7* | 2.73 | 3.21E-05 |
| YJL164C | *TPK1* | 1.75 | 5.33E-03 |
| YGL026C | *TRP5* | 2.02 | 9.31E-02 |
| YCR083W | *TRX3* | 4.10 | 1.34E-03 |
| DR513W | *TTR1* | 3.38 | 4.20E-02 |
| YBR082C | *UBC4* | 2.56 | 5.90E-02 |
| YPL072W | *UBP16* | 2.40 | 9.99E-02 |
| YGR184C | *UBR1* | 2.54 | 6.94E-02 |
| YDL058W | *USO1* | 2.36 | 3.16E-02 |
| YHR039C-A | *VMA10* | 6.91 | 2.19E-04 |
| YGR020C | *VMA7* | 1.85 | 7.09E-05 |
| YEL051W | *VMA8* | 2.56 | 9.03E-02 |
| YLL040C | *VPS13* | 4.18 | 1.05E-02 |
| YOR132W | *VPS17* | 3.84 | 7.90E-03 |
| YKR020W | *VPS51* | 1.98 | 8.56E-02 |
| YER072W | *VTC1* | 4.16 | 4.96E-03 |
| YPL252C | *YAH1* | 2.43 | 1.58E-02 |
| YLR200W | *YKE2* | 2.02 | 3.74E-02 |
| YKL196C | *YKT6* | 2.27 | 9.95E-05 |
| YFR049W | *YMR31* | 2.43 | 6.62E-02 |
| YDL235C | *YPD1* | 2.37 | 6.14E-05 |
| YML001W | *YPT7* | 3.51 | 1.57E-03 |
